# Infant Nephrogenic Diabetes Insipidus: Challenges Leading to Delayed Management

**DOI:** 10.1155/carm/2688113

**Published:** 2026-06-22

**Authors:** Sandra Stankovic M., Marija Jankovic Ratkovic, Danica Mihajlović, Milica Ignjatović, Milica Jakovljević, Vesna Cvetković, Dušan Miljković, Jelena Vučić, Danijela Jovančić Petković, Tatjana Stanković

**Affiliations:** ^1^ Department of Endocrinology, Pediatric Clinic, University Clinical Center, Nis, Serbia, ukctuzla.ba; ^2^ Medical Faculty, Nis, Serbia; ^3^ Department of Nephrology, Pediatric Clinic, University Clinical Center, Nis, Serbia, ukctuzla.ba; ^4^ Department of Neonatology, Pediatric Clinic, University Clinical Center, Nis, Serbia, ukctuzla.ba; ^5^ Department of Genetics, Pediatric Clinic, University Clinical Center, Nis, Serbia, ukctuzla.ba

**Keywords:** desmopressin partial response, hypernatremic dehydration, nephrogenic diabetes insipidus

## Abstract

**Background:**

Nephrogenic diabetes insipidus (NDI) is a rare, potentially life‐threatening renal tubular disorder characterized by a decreased ability to concentrate urine due to antidiuretic hormone (ADH) resistance, leading to significant free water loss. The congenital form, most often caused by X‐linked mutations in the AVPR2 gene, typically presents in early infancy. Due to its rarity and nonspecific clinical features in young children, such as failure to thrive, vomiting, and excessive fluid intake, diagnostic delays are common, especially in centers with limited prior experience in managing NDI.

**Case Presentation:**

We report a case of a 17‐month‐old male infant presenting with severe hypernatremia, dehydration, vomiting, and failure to thrive. Initial clinical findings, a partial response to desmopressin, and absent neurohypophysis signaling on MRI suggested central diabetes insipidus (CDI), leading to a delay in the correct diagnosis. However, persistent polyuria, despite escalating desmopressin doses, prompted reconsideration. Genetic testing confirmed a pathogenic AVPR2 mutation (c.541C > T, p. Arg181Cys), establishing the diagnosis of congenital NDI. Therapeutic management included thiazide diuretics, NSAIDs, and dietary sodium restriction, resulting in slow and gradual clinical stabilization.

**Conclusion:**

This case highlights several diagnostic challenges, such as misinterpreting partial desmopressin response and MRI results. It emphasizes the importance of systematic diagnostic algorithms for infants with polyuria and early genetic testing when clinical presentations lack clarity. We aim to share our clinical experience and the pitfalls encountered while managing a patient with NDI and contribute to earlier and more effective recognition and treatment of this rare condition, preventing the development of life‐threatening complications.

## 1. Introduction

Nephrogenic diabetes insipidus (NDI) is a rare but potentially life‐threatening renal tubular disorder characterized by a decreased ability to concentrate urine due to antidiuretic hormone (ADH) resistance, leading to significant free water loss [1–3]. Congenital forms, predominantly caused by mutations in the AVPR2 gene, are transmitted in an X‐linked recessive manner [4–6]. These mutations lead to dysfunctional vasopressin V2 receptors responsible for the kidneys’ response to ADH [2, 7]. This dysfunction typically presents during infancy or early childhood with polyuria, polydipsia, dehydration, and failure to thrive [1–3].

Its infrequent occurrence in routine clinical practice means that clinicians may only encounter this diagnosis sporadically, often limited to one or two patients throughout their professional careers [8]. Diagnosing NDI can be particularly difficult in young children, as the clinical presentation may overlap with other more common pediatric conditions [8, 9]. Consequently, managing such cases presents diagnostic and therapeutic challenges, potentially leading to delays in accurate diagnosis and the timely initiation of appropriate treatment [3, 10, 11].

Early recognition and accurate diagnosis are crucial. Delays in management could lead to severe complications such as severe dehydration, hypernatremia, and permanent neurological damage, particularly in young children [2, 12].

We aim to share our clinical experience and the pitfalls encountered while managing a patient with NDI and contribute to earlier and more effective recognition and treatment of this rare condition, preventing life‐threatening complications and improving long‐term outcomes [8, 12, 13].

## 2. Case Report

We present a case report of a 17‐month‐old male infant with NDI. The patient was transferred from a regional hospital due to profound hypernatremic dehydration occurring during an episode of intercurrent aphthous stomatitis. He presented with marked hyperpyrexia up to 40°C, vomiting, and significant failure to thrive. The patient was born prematurely at 34 weeks of gestation with a body mass of 2500 g. He was born as the third child into a family with a low socioeconomic status, which may have influenced the timing and quality of his medical care after two healthy girls. The mother reported that he had exhibited increased fluid intake since birth but without prior episodes necessitating hospitalization.

The patient presented with a significantly reduced body weight of 4.5 kg and a body height of 67 cm, corresponding to a weight/height ratio of −10 standard deviations (SDs). His length was markedly diminished, measuring 10 cm below the 3rd percentile (P3) for his sex and age. Also, his weight was profoundly reduced, registering 3.2 kg below P3 for his sex and age. Intellectual development was age‐appropriate, but significant motor delay was present, including the inability to sit independently.

The infant was admitted to the intensive care unit (ICU) exhibiting severe hypernatremic dehydration, with an initial serum sodium concentration of 169 mEq/L and low urine osmolality of 175 mOsmol/L with a remarkably increased urine output, ranging from 10 to 12 mL/kg/hour, despite a concurrent plasma arginine vasopressin (ADH) level of 4.5 pg/mL, which fell within the normal range (2–12 pg/mL). Volume resuscitation was aggressively pursued through the administration of hypotonic intravenous fluid volumes to address the excessive urinary loss (> 10 mL/kg/hour) and gradually correct hypernatremia. Comprehensive biochemical analysis revealed discrete elevations in serum urea and creatinine, while other parameters, including potassium, calcium, phosphate, alkaline phosphatase, and magnesium levels, were within normal limits. Furthermore, renal ultrasonography revealed no significant abnormalities.

Magnetic resonance imaging of the pituitary gland failed to demonstrate the neurohypophyseal signal.

Given the patient’s age, pronounced hypernatremia, and low urine osmolality, a formal water deprivation test was deemed unnecessary [1, 2]. To elucidate the underlying etiology of the polyuria and hypernatremia, a sublingual desmopressin test utilizing 30 μg of desmopressin acetate (DDAVP) in a Minirin Melt tablet formulation (a synthetic analog of arginine vasopressin) was performed to differentiate between central diabetes insipidus (CDI) and NDI. Following the initial administration of DDAVP, a discernible increase in urine density from 1.000 to 1.020 and a reduction in urine output to 7 mL/kg/hour were observed within 12 hours. This initial response suggested a diagnosis of CDI, prompting the initiation of DDAVP therapy at a dosage of 7 μg/day (Minirin Melt tablet) [9, 14]. However, subsequent monitoring revealed a persistent high urine output, maintained at 10–12 mL/kg/hour, with a consistently low urine density of < 1.003, despite a gradual escalation of DDAVP doses up to 120 μg/day over the ensuing four days [13, 14].

Due to the persistent polyuria, despite escalating DDAVP, the desmopressin was discontinued. A therapeutic regimen for presumed NDI was initiated, comprising ibuprofen at a dosage of 20 mg/kg/day, hydrochlorothiazide at a dosage of 2 mg/kg/day, and the administration of a hypo‐osmolar formula [8, 11]. Following the change in management, a gradual decrease in urine output from 10 mL/kg/hour to 7.2 mL/kg/hour was noted, with serum sodium levels progressively stabilizing within the 135–150 mEq/L range over the next 6 weeks.

Subsequent genetic analysis identified a specific pathogenic variant (c.541C > T p. (Arg181Cys)) in the AVPR2 gene.

No written consent has been obtained from the patients as there are no patient identifiable data.

## 3. Discussion

This case underscores the diagnostic and therapeutic dilemmas in managing congenital NDI, particularly when initial clinical features overlap with those of CDI [9–11]. The classic triad of polyuria, polydipsia, and hypernatremia can be particularly misleading in infants. A partial and transient response to desmopressin in our patient initially suggested a central etiology, contributing to a diagnostic delay [13, 14]. However, subsequent genetic testing identified a pathogenic AVPR2 mutation, confirming congenital NDI [4–6].

Although partial responsiveness to desmopressin in NDI is uncommon, it can arise from residual V2 receptor function, nonspecific stimulation of alternative vasopressin receptor subtypes, or renal adaptations secondary to chronic polyuria [3, 13, 14]. Recognizing these mechanisms is essential to prevent misinterpretation of an incomplete therapeutic response and premature exclusion of NDI from the differential diagnosis [3, 8, 13].

The absence of the posterior pituitary bright spot on a T1‐weighted MRI is typically considered a hallmark of CDI and must be interpreted cautiously. In NDI, chronic stimulation of vasopressin secretion may cause depletion of neurohypophyseal stores, which may also account for this finding [10]. This underscores the necessity of integrating imaging results with clinical and biochemical data [3, 9].

**FIGURE 1:**
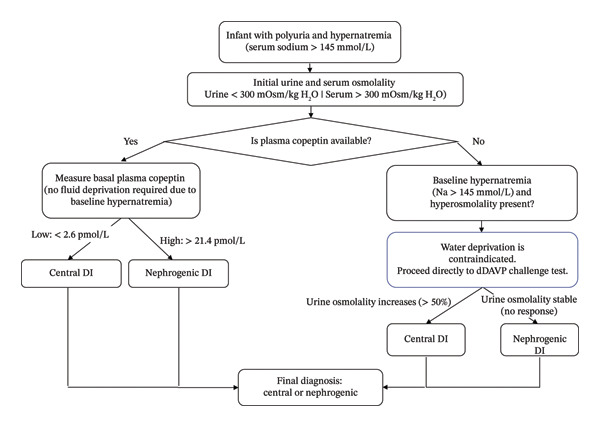
Proposed diagnostic algorithm for infants presenting with polyuria, hypernatremia, and suspected diabetes insipidus.

This case illustrates the importance of standardized diagnostic pathways for evaluating pediatric polyuria and hypernatremia, even in centers where NDI is perceived as exceptionally rare [3, 8]. Recently, plasma copeptin has emerged as a promising diagnostic biomarker in the evaluation of diabetes insipidus. As a stable surrogate marker for vasopressin secretion, copeptin measurement may help differentiate nephrogenic from CDI, particularly in infants where water deprivation testing may be unsafe and access to genetic testing is limited (Figure 1). Although copeptin measurement was unavailable in our center at the time of diagnosis, its implementation could potentially facilitate earlier recognition of congenital NDI in diagnostically challenging cases [15].

Our patient is the first confirmed case of NDI treated at our tertiary center in more than 30 years, even though we serve a population of two million people. This fact has contributed to diagnostic uncertainty and delayed management, highlighting the vulnerability of rare disease presentations in everyday clinical practice [8].

Therapeutically, achieving stabilization required a multimodal approach, including thiazide diuretics, NSAIDs, dietary sodium restriction, and meticulous fluid management [3, 8, 11]. Recurrent dehydration emphasized caregiver education and long‐term follow‐up, underlining the need for comprehensive patient care [8, 11].

By sharing this experience, we aim to improve the understanding of pediatric NDI. Increased awareness and sharing of such experiences are crucial for early recognition, faster diagnosis, and better management of patients with rare but life‐threatening conditions.

## Funding

No funding was received for this manuscript.

## Conflicts of Interest

The authors declare no conflicts of interest.

## Data Availability

The data that support the findings of this study are available on request from the corresponding author. The data are not publicly available due to privacy or ethical restrictions.
